# A maximum likelihood approach to generate hypotheses on the evolution and historical biogeography in the Lower Volga Valley regions (southwest Russia)

**DOI:** 10.1002/ece3.282

**Published:** 2012-07

**Authors:** Evgeny V Mavrodiev, Alexy P Laktionov, Nico Cellinese

**Affiliations:** 1 Florida Museum of Natural History, University of FloridaGainesville, Florida 32611–7800, USA; 2 Department of Biology, Astrakhan State UniversityAstrakhan 414056, Russian Federation

**Keywords:** Biogeography, Lower Volga Valley, mapping reconstruction, maximum likelihood, Russian flora, species stochastic, taxon-area analysis

## Abstract

The evolution of the diverse flora in the Lower Volga Valley (LVV) (southwest Russia) is complex due to the composite geomorphology and tectonic history of the Caspian Sea and adjacent areas. In the absence of phylogenetic studies and temporal information, we implemented a maximum likelihood (ML) approach and stochastic character mapping reconstruction aiming at recovering historical signals from species occurrence data. A taxon-area matrix of 13 floristic areas and 1018 extant species was constructed and analyzed with RAxML and Mesquite. Additionally, we simulated scenarios with numbers of hypothetical extinct taxa from an unknown palaeoflora that occupied the areas before the dramatic transgression and regression events that have occurred from the Pleistocene to the present day. The flora occurring strictly along the river valley and delta appear to be younger than that of adjacent steppes and desert-like regions, regardless of the chronology of transgression and regression events that led to the geomorphological formation of the LVV. This result is also supported when hypothetical extinct taxa are included in the analyses. The history of each species was inferred by using a stochastic character mapping reconstruction method as implemented in Mesquite. Individual histories appear to be independent from one another and have been shaped by repeated dispersal and extinction events. These reconstructions provide testable hypotheses for more in-depth investigations of their population structure and dynamics.

## Introduction

Biogeography as a synthetic discipline emerged because of the need to understand spatial patterns of biological diversity (Lomolino et al. 2006). From Darwin and Wallace through most of the 20th century, form and space were at the epicenter of the field. As phylogenetic studies became pivotal for inferring biogeographical hypotheses, the connection between patterns and their processes was still concealed by the lack of temporal information (Donoghue and Moore 2003). More recently, we have been able to include time in bio-geographic analyses due to the development of modern methods that better integrate fossil and molecular data (Sanderson 1997, 2002; Rambaut and Bromham 1998; Thorne et al. 1998; Cutler 2000; Huelsenbeck et al. 2000; Kishino et al. 2001; Thorne and Kishino 2002). Additionally, new methods for estimating ancestral area reconstruction in a temporal framework allow us to answer questions about the historical biogeography of individual lineages (Ree et al. 2005; Ree and Smith 2008) by integrating information about time of lineage divergences, geological history of the areas, and other relevant biological data (e.g., dispersal mechanisms).

Given all of the above, in many cases phylogenetic data are still very incomplete or completely lacking, although knowledge of the organisms living in an area and the history of the area is available. Therefore, in the absence of molecular phylogenies and temporal information, can we still generate testable hypotheses on the historical biogeography of an area and its organisms using modern approaches? We propose a study where species occurrence data in conjunction with well-known abiotic parameters may still inform the biogeographical history of an area and the individual taxa that inhabit it. Our approach is based on distribution data alone when this is the only available information that can be used in a biogeographic analysis, when the only alternative would be to perform no analyses. The information inherent to floristic data is clearly limited, but may still allow a first attempt to generate viable hypotheses on species history that can be further tested with more appropriate methods or when more data become available.

The Volga is Europe's longest and largest river and an important feature in Russia's landscape, culture, and bio-diversity. It rises in the Valdai Hills, northwest of Moscow, and runs 3530 km across southeastern Russia to finally empty in the Caspian Sea, making it the largest river that does not drain into an ocean (Kroonenberg et al. 1997). The Caspian Sea, in turn, is the largest continental water body on Earth and has over 80% of water inflow from the Volga River (Dumont 1998). Currently at –26.3 m below global sea level, this large basin has a complex history characterized by tectonic movements caused by the closing of the Thethys Sea, and several transgression and regression events that are deeply associated with the overall formation of the Low Volga region and its biodiversity (Kroonenber et al. 1997; Mamedov 1997; Dumont 1998).

The genesis of the modern Volga River and its Delta dates back from the Pliocene when glaciation cycles have largely contributed to a series of dramatic transgression events that culminated with the Late Khvalyn phase (the last major transgression in the Pleistocene) and were intercalated by regression events that include the current Novocaspian phase (Kroonenberg et al. 1997; Mamedov 1997; Dumont 1998). Although the sequence of events is not controversial, the actual chronology is still unclear and debated in the literature despite the numerous ^14^C dates generated by various studies (Kaplin et al. 1993; Kroonenberg et al. 1997; Mamedov 1997; Dumont 1998). Overall, the last transgression event, with all of its phases, may have occurred approximately within 16–7 ka ago (Svitoch 2008), although older dates are provided by Kaplin et al. (1993), Kroonenberg et al. (1997), Mamedov (1997), and Dumont (1998).

**Figure 1:**
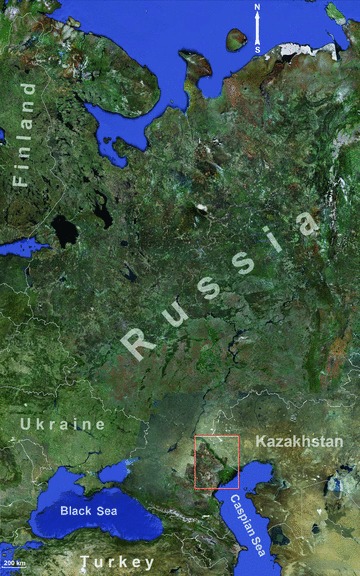
Lower Volga Valley indicated by the area within the inset.

The Astrakhan region comprises the Volga River's southern most part (Fig. 1) with 13 distinct local floristic areas that include Mountain Bolshoe Bogdo and Lake Baskunchak in the northeast (reviewed in Popov 2004; A. V. Popov, unpubl. data) and 11 additional areas forming the Lower Volga Valley (LVV) (Golub et al. 2002; Laktionov 2003; Laktionov and Afanasiev 2007; Losev et al. 2008). The LVV is about 500 km long and nearly 1.5 million sq. km and comprises two parts, namely the Volga-Akhtuba floodplain and the Volga Delta (Golub and Mirkin 1986). As broadly defined (Golub et al. 2002; Laktionov 2003), the LVV includes adjacent steppe and semidesert areas. Along the arid Caspian lowlands, the valley is remarkable for its vegetation diversity and includes swamps, meadows, fens and forests, whose overall biological productivity is considerably superior to that of zonal xerophytic communities (Golub and Mirkin 1986). Generally, the floodplain is characterized by high concentration of salts that reaches its maximum levels in salt marshes where only halophytes can grow. However, when covered with fresh water, salts are carried away giving rise to less hostile ecosystems and a variety of vegetation structures (Shein et al. 2011).

Fursajew (1937, 1940) indicated that the level of endemism and general diversity of the LVV is essentially underestimated. He found a significant number of undescribed taxa in several traditionally circumscribed genera including *Alopecurus*, *Althaea*, *Astragalus*, *Bromopsis*, *Carex*, *Corispermum*, *Elytrigia*, *Glyceria*, *Hierochloe*, *Lotus*, *Phalaris*, *Plantago*, *Poa*, *Polygonum*, *Rorippa*, *Salix*, *Senecio*, *Setaria*, *Simphytum*, *Thalictrum*, *Trapa*, and *Veronica* among many others (see also the numerous Fursajew collections deposited in the herbarium of Saratov State University [SARAT]). This large number of taxa unknown to science was probably due to recent and rapid evolution in the LVV area, most likely caused by selective responses to the numerous Volga floods and more recent transgression processes that took place since the Pleistocene (Fursajew 1937, 1940). Some of these taxa were later formally described by other botanists. According to Fursajew (1937, 1940), the LVV is a highly dynamic area that contains many more unknown endemics than recognized thus far, and may actually represent a biodiversity hot spot in southeast Eurasia that clearly requires more investigation. More evidence of the LVV high diversity is provided by the Herbarium of Otto Kuntze who traveled from Sarepta (modern Volgograd) to Astrakhan in 1886 during his famous Caucasian trip, and collected and described many new taxa (Zanoni 1980; Zanoni and Schofield 1981).

Over the past 12 years, we gathered a large amount of detailed floristic data covering 13 local floristic regions that characterize the LVV (Laktionov 2003; Laktionov and Afanasiev 2007; Losev et al. 2008; Laktionov 2009; A. P. Laktionov, unpubl. data). Specifically, we recorded the occurrence of 1018 species representing nearly all of the known LVV local flora, and including 23 endemic species restricted to one or few of the 13 floristic regions. The goal of this study is to infer the putative history of the areas comprising the LVV and reconstruct the individual histories of each species in order to generate hypotheses on the general processes affecting the LVV in view of its known complex geological history.

Taxon-area analyses have dominated primarily the field of cladistic biogeography and have been, and still are, extensively used in approaches such as parsimony analysis of endemicity (PAE) and its several methodological variations (Morrone and Crisci 1995). Cladistic biogeography and PAE have been criticized in the literature, particularly due to lack of integrated temporal information leading to significant misinterpretation of the connection between patterns and their causal processes (Donoghue and Moore 2003). However, general PAE approaches are still used (Porzecanski and Cracraft 2005; Contreras-Medina et al. 2007; Escalante et al. 2009, among many others), regardless of old and new critiques (Garzon-Orduna et al. 2008). We are fully aware and acknowledge the limitations of PAE for not laying its methodological foundations on phylogenetic or temporal information, and de-emphasizing dispersal and extinction as likely causal processes. In this study, we adopt the basic assumption that through modern taxon distribution alone we may still recover a strong historical signal about area relationships (Cracraft 1991; Porzecanski and Cracraft 2005); however, rather than using maximum parsimony (MP), we choose to analyze our data in a maximum likelihood (ML) framework. In PAE, synapomorphies are usually interpreted as vicariance events, parallelism and convergence as dispersal events, and reversals as extinctions (e.g., Morrone and Crisci 1995). ML provides a model-based approach in which area relationships are explained based on the degree of likelihood that species are present on each node of the taxon-area phylogeny. No preferred causal process is evoked and vicariance, dispersal, and extinction become equally possible. Moreover, in contrast to PAE, all taxa, including widespread and narrow endemics, are considered informative and the history of each species can be individually reconstructed on the ML topology, allowing us to generate testable hypotheses based on their putative biogeography.

## Materials and Methods

### Areas and taxa

**Figure 2:**
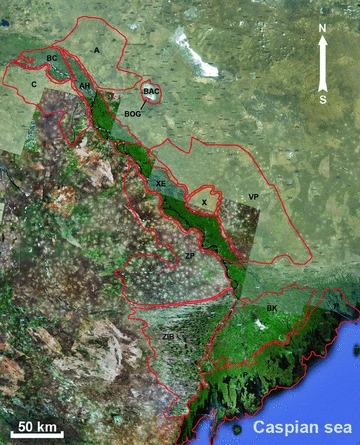
Map showing the 13 floristic areas. BOG, Mountain Bogdo; BAC, Lake Baskunchak; VP, Dosang; ZP, Jenotaevka, Raccoons Village; X, Tambov Village or Harabaly; C, Ulmus Village; A, Cabbage's Ravine, Kapoostin Yar; P, Three House Village; AH, Kozinka; BC, Garden Place; XE, Carp Borough; ZIB, Lakeland; BK, Blue-grey Hill.

Our original investigation of the LVV (including adjacent areas) based on floristic and abiotic data lasting 12 years, helped to define 13 distinct floristic regions (as summarized in Laktionov and Afanasiev 2007) (Fig. 2): (1) BOG: Mountain Bogdo (Rus.: “гора” Большой Богдо); (2) BAC: Lake Baskunchak (Rus.: озеро Баскунчак); (3) VP: Dosang (Rus.: Досанг); (4) ZP: Jenotaevka, Raccoons Village (Rus.: Енотаевка); (5) X: Tambov Village or Harabaly (Rus.: Тамбовка, Харабали); (6) C: Ulmus Village (Rus.: Вязовка); (7) A: Cabbage's Ravine, Kapoostin Yar (Rus.: Капустин Яр); (8) P: Three House Village (Rus.: Трехизбинка); (9) AH: Kozinka (Rus.: Козинка); (10) BC: Garden Place (Rus.: Садовое); (11) XE: Carp Borough (Rus: Сазаний угол); (12) ZIB: Lakeland (Rus.: Озерное); and (13) BK: Blue-grey Hill (Rus.: Сизый Бугор).

Garden Place (BC), Kozinka (AH), and Carp Borough (XE) strictly represent the river valley; Three House Village (P) includes the narrowly defined river delta along with the small islands off the Caspian coastline; Blue-grey Hill (BK) and Lakeland (ZIB) are components of the more widely defined river delta and contain the chains of Baer knolls; the dry steppe and desert areas of Cabbage's Ravine (A), Ulmus Village (C), Dosang (VP), Jenotaevka (ZP), and Tambov Village (X) are immediately adjacent to the valley forming the extended LVV (Fig. 2). The old and geomorphologically unique Lake Baskunchak (BAC) and Mountain Bogdo (BOG) are salt formations located in the northeastern portion of the valley but still included in the study area. These local floristic areas have also been postulated and described in details by Laktionov (2003), Losev et al. (2008), and Laktionov and Afanasiev (2007), partly based on Fursajew (1940), Golub et al. (2002), and Popov (2004; A. V. Popov, unpubl. data). All 13 areas are distinct in habitat and floristic composition and may contain from few (e.g., X and XE) to many (e.g., BK) single-site endemic species.

Species occurrence data was based on herbarium collections and fieldwork. Specimen data were collected from the herbaria of the following Institutions: Astrakhan State University (AGU), Saratov State University (SARAT), Volgograd State Pedagogical University (VOLG), Institute of Ecology of the Volga Basin, Russian Academy of Sciences (TLT), Biological and Geographical Departments of M. V. Lomonosov Moscow State University (MWG and MW), Main Botanical Garden of Russia (MHA), and Komarov Botanical Institute, Russian Academy of Sciences (LE). The complete floristic treatment of Mountain Bogdo and Lake Baskunchak areas has been the focus of Popov's recent studies (2004; A. V. Popov, unpubl. data) and is in its final stage of preparation. Therefore, for the purpose of our study we used his preliminary list of taxa.

We recorded the occurrence of 1018 species (see Appendix S1) across the broadly defined LVV, representing the comprehensive regional flora (Laktionov 2009; A. P. Laktionov, unpubl. data) but excluding all elements that we believed are non-native to the areas and potential recent introductions (alien species). Additionally, we disregarded any anthropogenic areas or localities that were significantly disturbed.

Nomenclature in this study follows Cherepanov (1995). Based on our taxon sample and well-defined areas, we constructed a taxon-area matrix (Appendix S1).

### Taxon-area analyses

ML analysis using RAxML (Stamatakis 2006) was used to reconstruct the relationships of floristic LVV areas. The simple Mk model with gamma distribution of rate of hetero-geneity (G) was selected. The Mk model was originally proposed by Lewis (2001) as a generalized Jukes and Cantor model (Jukes and Cantor 1969) to be implemented for analyzing discrete morphological data. The model assumes that a lineage is always in one of *k* possible states (≥2) with no state considered plesiomorphic or apomorphic (Lewis 2001). Additionally, character states can freely change along any branch at any time, with the probability of each change occurring equally during all such time intervals along the branches. Therefore, in our particular case, changes from species absence (0) to species presence (1) and vice-versa are all equally probable. Lewis (2001) acknowledges that this model may be considered unrealistic and discusses in details the reasons why it is very appropriate in discrete character-based analyses. The model was selected here because it provides the simplest assumption to statistically reconstruct the LVV area relationships using discrete characters, and it may help generating more realistic hypotheses than parsimonious reconstructions. This approach has been applied to recent biogeographical studies using species distribution data (Vanderpoorten et al. 2007, 2010).

In addition to using the gamma substitution model (G), we analyzed our data by selecting the G + I model to account for the presence of invariant sites and obtained the exact same results.

As a comparison, MP analysis was also conducted with PAUP* 4.0b10 (Swofford 2002), using heuristic searches with 1000 random addition replicates, with no more than 100 trees saved per replicate, and tree-bisection-reconnection (TBR) branch swapping with the MulTrees option in effect. All characters were treated as unordered (Fitch Parsimony) and no weighting procedure was performed (Fitch 1971). Bootstrap analyses were conducted with 2500 replicates for both MP and ML.

All analyses included an all-zero hypothetical artificial outgroup for rooting purpose (Rosen 1988; Cracraft 1991; Morrone 1994; Trejo-Torres and Ackerman 2001; Porzecanski and Cracraft 2005). However, as an alternative approach, we also used Lundberg rooting (Lundberg 1972) where the area with the least number of taxon occurrence is used as outgroup (hypothetical ancestral area). In this study, Mountain Bogdo (BOG), which happens to represent the oldest geo-morphological area among all investigated (Popov 2004), represents the area with the least number of species occurrence and was used to root the tree. In addition to MP and ML, we used a more traditional clustering approach by performing a UPGMA analysis to assess overall area similarity based on species occurrence but independently from common ancestry.

In ML analyses of binary datasets character absences are equally informative as character presences; consequently, all-zero columns provide as much information as all-one columns. In the context of our approach, this means that we can estimate the history of floristic areas in the LVV based not only on extant species occurrence, but also on a hypothetical palaeoflora. Therefore, in addition to the analyses described above we generated a matrix that included various numbers of hypothetical extinct taxa (all-zero columns). Essentially, we assumed that the dramatic transgression and regression events that occurred throughout the Pliocene and Pleistocene caused mass extinction but in the absence of essential knowledge about the past ancestral flora, we simulated a number of scenarios that featured 509, 1018, 1527, and 2036 extinct taxa. We created matrices that included: 509 extinct taxa (509 all-zero columns included) representing half the number of the species in the extant flora; 1018 extinct taxa (1018 all-zero columns included) that assumed an equal number of ancestral species to the extant flora; 1527 extinct taxa (1527 all-zero columns included) that assumed an ancestral flora larger by half the size of the extant flora; and 2036 extinct taxa (2036 all-zero columns included) assuming an ancestral flora twice as big as the extant number of species. Felsenstein (1992) introduced a correction for the omission of absent locations in the matrix and proposed to compensate by adding to the data a fictional location 0 at which all species lack the recognition sequence. Indeed, given the potential effect on the transition rates generated by adding many zero-columns, we wanted to assess how these would affect the topology.

Matrices were analyzed using the same ML approach described above but with an all-zero hypothetical outgroup. In our specific scenarios, we assumed that the multiple transgression events may have likely led to the extinction of the ancestral flora; therefore, there is no contextual difference between the functional outgroup (all-zero column) and the extinct taxa. In other potential scenarios, all-zero columns could also represent failure to colonize an area.

Individual taxon histories were recovered using stochastic character (species) mapping reconstruction (Nielsen 2002; Huelsenbeck et al. 2003). Although MP often makes reasonable reconstructions of the history of a character, it has a number of limitations including the inability to consider more than a single change along a branch on the tree and the uncoupling of evolutionary time from the number of character changes (Huelsenbeck et al. 2003). In other words, stochastic mapping reconstruction depicts character states at the nodes and along all branches of the tree, allowing predictions of character changes at any point in the history of a taxon. We reconstructed species histories using an approach originally described by Nielsen (2002), extended by Huelsenbeck et al. (2003), and implemented in Mesquite (Maddison and Maddison 2010). Species histories are modeled as a continuous-time Markov chain where all possible changes along the taxon-area tree are considered. As an example, we will show the advantages of reconstructing species history using stochastic mapping versus MP (see Results).

## Results

### Area relationships and overall similarity

**Figure 3:**
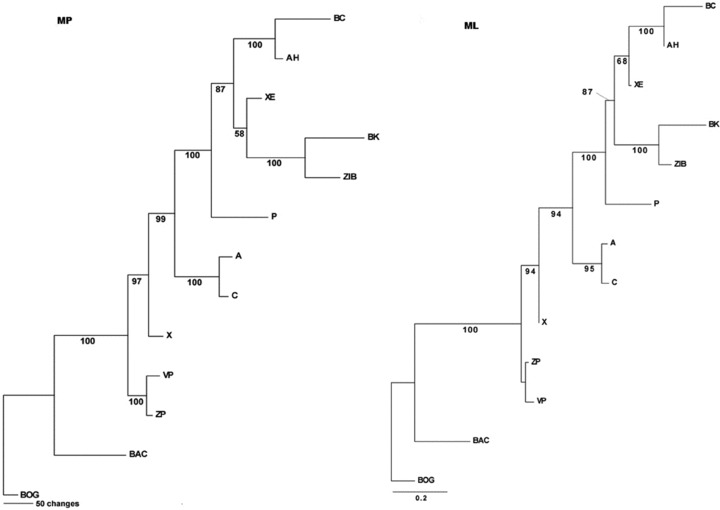
Maximum parsimony (a) and maximum likelihood (b) phylogenies of 13 floristic areas estimated based on species composition (Lundberg rooting). Tree statistics: (a) length = 1576, CI = 0.607, RI = 0.662; (b) Log value: −ln L = 5730.608282.

**Figure 4:**
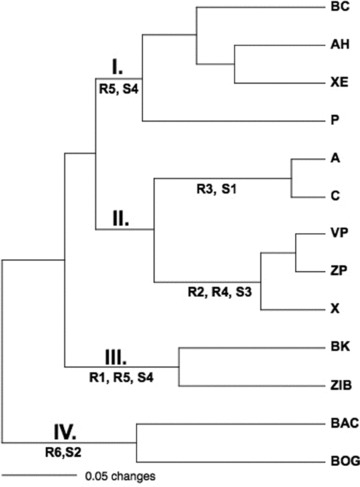
UPGMA dendrogram. Numbers above branches: I. Delta and River Valley s. str.; II. Dry Steppe and Desert; III. Baer Knolls; IV. Lake Baskunchak and Mountain Bogdo. Numbers below branches indicate the major types of reliefs and soils: R1 New Caspian accumulative sea plain; R2 Upper Khvalyn accumulative sea plain; R3 Lower Khvalyn accumulative sea plain; R4 Atmogenic sea plain; R5 Alluvial plain; R6 Relief caused by denudation. S1 Chestnut-colored saline soils and clay-based substrates; S2 Alkaline soil and salt marshes; S3 Sandy saline soils, sands, and sandy loams; S4 Different types of alluvial-based soils. See Atlas of the Astrakhan Region (1997) for more details.

MP and ML analyses each generated a single tree (Fig. 3a and b). Within each analytical approach (MP and ML), rooting with an artificial ancestral area (all-zero outgroup) or using Mountain Bogdo (BOG) as the hypothetical ancestral area (Lundberg rooting) generated identical results. The ML and MP topologies are congruent, except that in the MP tree Carp Borough (XE) is sister to the clade that includes Lakeland (ZIB) and Blue-grey Hill (BK), whereas in the ML tree area XE is sister to the clade including Kozinka (AH) and Garden Place (BC). Support values for all clades are strong across the topologies. Mountain Bogdo (BOG) is sister to the rest of the tree regardless of rooting options (Fig. 3). The adjacent Lake Baskunchak (BAC) is recovered as sister to the rest of the areas. Dry-steppe/semidesert areas such as Dosang (VP), Jenotaevka (ZP), Tambov Village (X), Cabbage's Ravine (A), and Ulmus Village (C), although similar in floristic composition (Fig. 4), do not form a clade. Clades are formed by Dosang (VP) and Jenotaevka (ZP), and Cabbage's Ravine (A) and Ulmus Village (C), located across from each other on opposite sides of the valley and with similar geomorphological history. Area X is sister to a large clade that includes all the rest of the components forming the Lower Volga River Valley (Fig. 3).

UPGMA analyses yielded a dendrogram with the clusters defined by soil type and overall ecosystem (Fig. 4). Garden Place (BC), Kozinka (AH), Carp Borough (XE), and Three House Village (P) form the narrowly defined river valley and delta characterized by swamps. Cabbage's Ravine (A), Ulmus Village (C), Dosang (VP), Jenotaevka (ZP), and Tambov Village (X) are largely desert and steppe habitats with A and C having a clay and silt substrates and VP, ZP, and X mainly a sand basalt. Blue-grey Hill (BK) and Lakeland (ZIB) are characterized by the presence of the Baer knolls, which are shale formations between ca. 5 and 20 m in height, formed by the action of onshore winds and sea level fluctuations. Lastly, Lake Baskunchak (BAC) and Mountain Bogdo (BOG) feature extremely salty environments.

Matrices that included 509, 1018, 1527, and 2036 hypothetical extinct taxa generated topologies that are generally congruent with our previous results but differ in support values and relationships of some of the areas (Fig. 7). Adding only 509 extinct taxa (half the size of the extant flora), we obtain a cladogram with moderate to strong support recovering Dosang (VP), Jenotaevka (ZP), and Tambov Village (X) forming a gradient at the base of the topology; Three House Village (P) as sister to Blue-grey Hill (BK) and Lakeland (ZIB); and Carp Borough (XE) as sister to Garden Place (BC) and Kozinka (AH). A different scenario is generated by analyzing the matrix with an equal number of extinct taxa (1018). In this case, Cabbage's Ravine (A) and Ulmus Village (C) are at the base of the topology and Tambov Village (X) is recovered as sister to Dosang (VP) and Jenotaevka (ZP). Some clades have low to no support although these were previously recovered with higher support. Including 1527 and 2036 extinct taxa generates a topology that is very similar to the topology generated without the inclusion of extinct taxa and has Tambov Village (X) as sister to Dosang (VP) and Jenotaevka (ZP) with good to strong support values across both trees. All the above topologies feature a strongly supported clade that include Three House Village (P), Blue-grey Hill (BK), Lakeland (ZIB), Carp Borough (XE), Garden Place (BC), and Kozinka (AH), forming the narrowly defined river valley and the delta region. The sister relationship between Cabbage's Ravine (A) and Ulmus Village (C) is recovered in most, as is the relationship between Tambov Village (X), Dosang (VP), and Jenotaevka (ZP).

### Stochastic species mapping reconstruction

**Figure 5:**
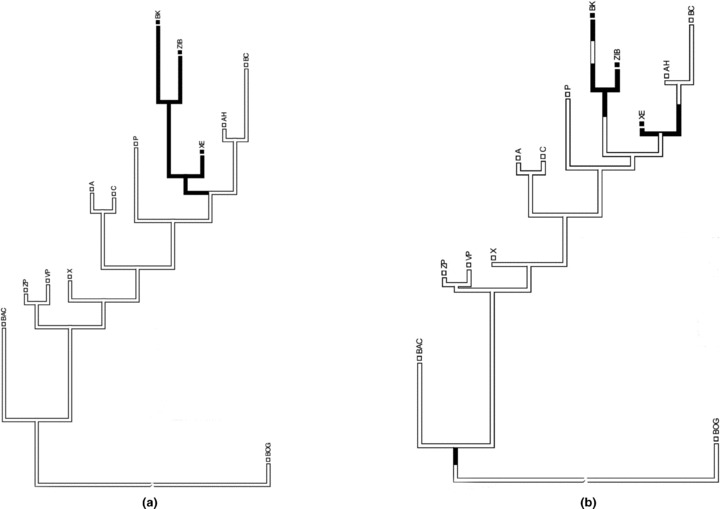
Maximum parsimony (a) and species stochastic mapping reconstruction (b) of *Crepis astrachanica* within the Lower Volga Valley.

**Figure 6:**
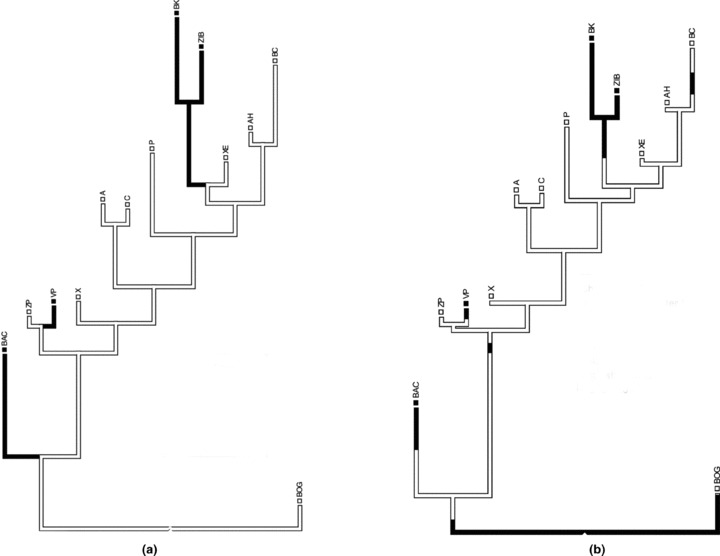
Maximum Parsimony (a) and species stochastic mapping reconstruction (b) of *Suaeda salsa* within the Lower Volga Valley.

Stochastic species mapping reconstructions for each of the 1018 species can be found in Appendix S1 by downloading a matrix that can be opened with the freely available Mesquite (Maddison and Maddison 2010). Every map represents only one of numerous possible species histories and each of these suggests a more complex scenario when compared to parsimony reconstructions (see also Discussion). For example, Figure 5 shows the reconstructions of *Crepis astrachanica,* a narrow LVV endemic, using MP (5a) and stochastic mapping (5b), respectively. MP simply suggests that *C. astrachanica* is an endemic that may have evolved in the ancestral areas of Blue-grey Hill (BK), Lakeland (ZIB), and Carp Borough (XE). However, a closer look into stochastic mapping reconstruction shows that its presence at the base of the cladogram suggests that this taxon may have been present in the entire ancestral areas that include Three House Village (P), Blue-grey Hill (BK), Lakeland (ZIB), Carp Borough (XE), Kozinka (AH) and Garden Place (BC), and may have subsequently become extinct in a few of these. Similarly, for the nonendemic *Suaeda salsa*, parsimony reconstruction (Fig. 6a) suggests that this species may have been present in the ancestral area of Blue-grey Hill (BK) and Lakeland (ZIB) where it still occurs and have dispersed into Dosang (VP) and Lake Baskunchak (BAC), or vice versa from BAC or VP into BK and ZIB. However, stochastic mapping re-construction shows a much more accurate putative history with the likelihood of this species occurrence in more nested ancestral areas (Fig. 6b). Given the more in-depth scenarios, we obtain from analyzing the stochastic reconstruction of each species, we will not discuss any further results obtained by MP.

## Discussion

### The history of the LVV floristic areas

The numerous Caspian transgressions that occurred since the Miocene and throughout the Pleistocene are the main processes that led to the formation of the LVV and its adjacent areas (Kroonenberg et al. 1997; Svitoch 2008; Badyukova 2010). However, there is little agreement regarding the chronology and paleogeographical details of the Caspian transgressions and how they affected the formation of various areas in the LVV (Kaplin et al. 1993; Krooneberg et al. 1997; Dumont 1998; Svitoch 2008).

The topology obtained by ML shows Mount Bogdo and Lake Baskunchak at the base of the tree. Mount Bogdo was never completely covered by water during the numerous Caspian transgressions, and with Lake Baskunchak represents the oldest geomorphological feature of the study area, dating back to the Oligocene (Popov 2004; A. V. Popov, unpubl. data). Aside from areas BOG and BAC, our results suggest that the floristic areas that include Dosang (VP) and Jenotaevka (ZP) represent the oldest of the broadly defined LVV (Fig. 3). However, previous studies have indicated that Kapoostin Yar (A) and Ulmus Village (C) are the first geomorphological formations after the last transgression of the Caspian Sea (Svitoch and Kluvitkina 2008). In other words, Dosang (VP) and Jenotaevka (ZP) formed after Kapoostin Yar (A) and Ulmus Village (C), and are mostly the result of the sand, semi-sand substrates left over by the last regression of the Caspian Sea (Svitoch and Kluvitkina 2008). We are aware that although branch length was determined by our analysis, we cannot easily infer the actual age of extant floristic regions. However, the ML topology (Fig. 3) clearly shows that the floristic ancestral areas of VP and ZP are older than the ancestral areas of A and C, regardless of their geo-morphological histories.

Intuitively, as the Caspian Sea slowly regressed southbound, the northernmost plains were indeed the first to form. However, based on our results, the northernmost areas were not necessarily the first to harbor new floristic elements. The history of the local flora may not correspond to the chronology of area formations. For example, our results suggest that the flora composition along the Volga River such as Kozinka (AH) and Garden Place (BC) not only is different from that of immediately adjacent areas (A and C) (Fig. 4, UPGMA), but it is also more recent (Fig. 3). If we only considered the transgression and regression events as they occurred in chronological order, we would have to assume that the floras in Kapoostin Yar (A), Ulmus Village (C), Garden Place (BC), and Kozinka (AH) were of similar age, as these areas clearly formed approximately at the same time. This incongruence suggests that the formation of the modern flora in the LVV started after the late Khvalyn transgression phase of the Caspian Sea, as well as subsequent minor events. Therefore, the flora of the Lower Volga River Valley and its adjacent areas represents a young entity, approximately 5000–7000 years old, and with the putative exclusion of Blue-grey Hill (BK) and Lakeland (ZIB), formed independently from the direct influence of the late Caspian transgressions.

Our findings are congruent with Fursajew's conclusions (1940). He suggested that the modern LVV flora formed only at the very end of the Khvalyn phase (Fursajew 1940). The time periods previous to the Khvalyn phase were characterized by the extinction of a paleo-Volga flora due to the multiple dramatic Caspian events followed by subsequent recolonization and local speciation in newly formed areas (Fursajew 1940). The large number of different families, each represented by a small number of species, may also be an indication of the young age of the flora (Laktionov 2009).

Interestingly, Tambov Village (X) and Kozinka (AH) appear to have no branch length (Fig. 3b). Branch length represents the number of changes in terms of taxon presence or extinction, essentially any change from 0 to 1 or 1 to 0. Therefore, short or no branch length may suggest that these areas still harbor their ancestral flora, or certainly enough elements that were once part of it. The nonzonal vegetation of AH is peculiar for its dominant willow (*Salix alba* s. l.) and poplar (*Populis nigra* s. l.) forests and multiple floodplain meadows of the “low” and “middle” levels (Fursajew 1940). These types of floodplain meadows are covered by water for at least one to two months annually and form different plant communities that include *Althaea officinalis, Carex acuta, C. melanostachya, Elytrigia repens, Galium verum, Hierochloe stepporum, Lythrum salicaria, Senecio jacobae,* various species of *Bolboschoenus, Bromus,* and *Eleocharis*. Many of these broadly defined taxa were proposed by Fursajew (1940) as represented in the LVV by unique “races” of different taxonomical rank. The vegetation of Tambov Village (X) is characterized by mostly sandy deserts and various xerophytic, semishrub communities including *Artemisia lerchiana*, *A. arenaria*, *Calligonum aphyllum,* and others, often in association with various shrubs (e.g., species of *Tamarix*) as well as annuals (e.g., *Agrophyllum squarrosum* and species of *Corispermum*).

The areas of Blue-grey Hill (BK) and Lakeland (ZIB), among the most recently formed in the LVV, are characterized by the presence of peculiar reliefs known as the Baer knolls chains (Svitoch and Kluvitkina 2008). These are symmetric, wave-like shale formations 5–25 m in height, 1–1.5 km wide, and usually few kilometers long, often arranged sublatitudinally and separated by depressions measuring 0.5–5 km or more (Shein et al. 2011). The knolls are dry elevations covered with sparse vegetation on a saline and nonsaline, semi-desert soils, but based on the alluvial substrates. Our results are in agreement with the hypothesis of a recent stadial-coastal origin of the Baer knolls, which were formed within a short period of time as the result of multiple transgression and regression events that led to fluctuations of the Caspian Sea level on the very late Khvalyn phase (Svitoch and Kluvitkina 2008; Mitina and Malashenkov 2009). Their recent formations seem to be the casual events that led to the separation of the Blue-grey Hill (BK) and Lakeland (ZIB) from the rest of the LVV.

Areas BK and ZIB are remarkable in their floristic composition combining elements of swamp and steppe/semi-desert flora. The steppe flora of the Baer knolls seems very different from the flora in other steppe areas in the LVV and was formed mostly as the result of multiple dispersal events. Fourteen of the 23 endemic species are present in both of these areas, and a few others in either BK or ZIB. The formation of the Baer knolls may have contributed to high level of endemism of the areas BK and ZIB.

**Figure 7:**
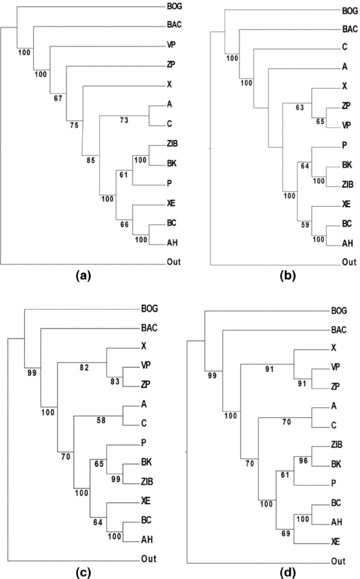
Maximum likelihood analyses of taxon-area matrices including 509 (a), 1018 (b), 1527 (c), and 2036 (d) hypothetical extinct taxa. Log values: a. −ln L = −6934.304102; b. −ln L = −7387.621514; c. −ln L = −7714.979075; d. −ln L = −7973.271501.

Adding to the above scenario, we considered the occurrence of a hypothetical palaeo flora that became extinct due to those dramatic geological activities that led to the many transgression and regression events. In the absence of significant paleobotanical data, we performed analyses that included putatively different sizes of a paleoflora (Fig. 7a–c). All four topologies obtained with the inclusion of different hypothetical numbers of extinct taxa recovered scenarios that are similar, although not completely congruent with the scenario recovered from the analysis solely based on extant taxa. The analysis including 1018 extinct and extant species suggests that Cabbage's Ravine (A) and Ulmus Village (C) are floristically older than other areas along the river valley (Fig. 7b). This hypothesis is the least supported and suggests that the evolution of the flora is consistent with the chronology of the areas formation. However, in the remaining three simulations, (Fig. 7 a, c, and d) the sand-based areas of Dosang (VP), Jenotaevka (ZP), and Three Houses Village (P) appear older than the rest of the narrowly defined LVV areas. In all simulations, Three Houses Village (P) is sister to the Baer knolls areas (ZIB and BK), and Carp Borough (XE) is sister to Lakeland (ZIB) and Blue-grey Hill (BK). These two clades are consistently recovered as forming the strict Volga River valley.

Our results show that regardless of the inclusion of hypothetical extinct taxa, we consistently recover a river valley and delta regions that harbored flora only after some of the adjacent areas were colonized. Incongruence is recovered only with regard to the relationships of Dosang (VP) and Jenotaevka (ZP), Cabbage's Ravine (A) and Ulmus Village (C) and their respective positions in the topology. However, there seems to be a general consensus for a flora that evolved first in Tambov Village (X), Dosang (VP) and Jenotaevka (ZP), following later in Cabbage's Ravine (A) and Ulmus Village (C), and finally all across the river valley and delta. The relationship of Tambov Village (X), Dosang (VP) and Jenotaevka (ZP) is also supported by the notion that these areas are largely desert steppes on mainly sand basalt substrates. Similarly, Cabbage's Ravine (A) and Ulmus Village (C) are characterized by similar dry habitats on mainly clay and silt substrates (Fig. 4, UPGMA).

### Stochastic species mapping reconstructions

**Figure 8:**
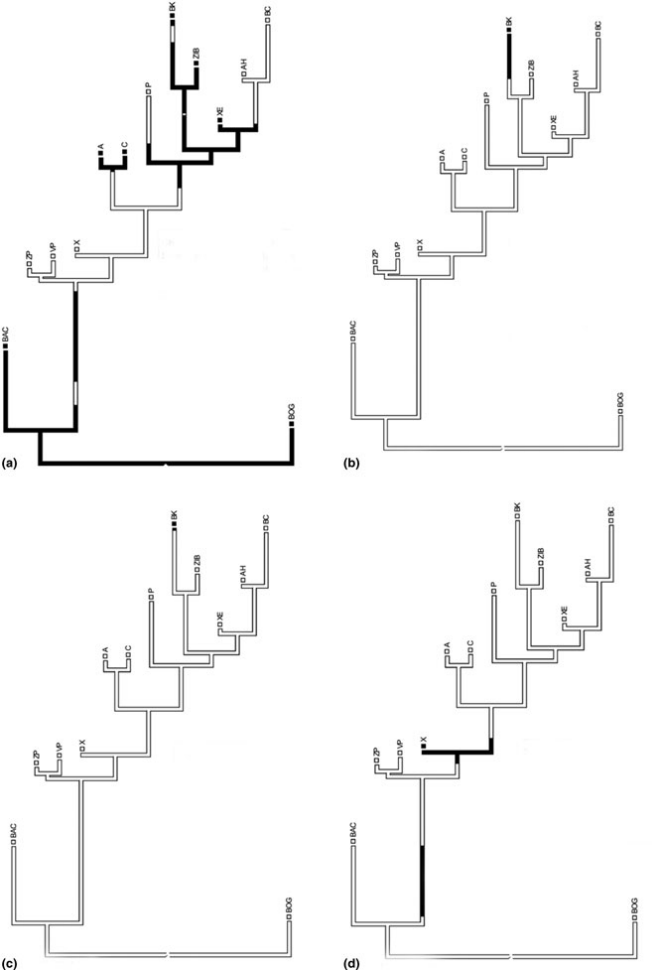
Species stochastic mapping reconstruction of selected species within the Lower Volga Valley: *Artemisia astrachanica* (a), *Astragalus baerii* (b) *Ceratophyllum kossinskyi* (c), *Corispermum filifolium* (d).

Given the complex scenarios characterized by a geologically dynamic active region, the history of the LVV flora clearly reflects the ramifications of these activities. We examined the ancestral reconstruction of each species along the branches and nodes of the taxon-area tree to generate testable hypotheses that may prompt future in-depth analyses on the population structure and dynamics of individual species. The current species distribution may harbor historical complexity that is likely tied to the geological activities in the Caspian Sea and LVV. Figure 8a shows the reconstruction of *Artemisia astrachanica*, an endemic species currently found in extremely salty environments (BAC and BOG), on clay-silty substrates (A and C), along the swamps of the river valley (XE), and on the dry Baer knolls (ZIB and BK). The reconstruction also shows that this species was present in the ancestral areas of the LVV but never successfully established in mostly sandy and dry steppes environments (VP, ZP, and X), despite the ability of this species to grow on sandy, salty semi-meadow substrates, also known as limans (e. g., Boonman and Mikhalev 2005). Extinction may explain its absence from part of the river delta (P), whereas its presence in areas A and C maybe have been caused by dispersal from either swamps (XE) or salty environments (BOG and BAC). Therefore, the presently disjunct distribution of *A. astrachanica* may be explained by both dispersal and extinction events across the valley. Even in area BK we notice that current populations may not necessarily be the same populations that had previously occupied this areas. Similarly, dispersal events from clearly older, salty substrates into the river valley, followed by subsequent dispersal and/or extinction may have shaped the current distribution of several other endemic species (see reconstructions in Appendix S1: *Apocynum kazakevichii nom. provis., Centaurea astrachanica, Euphorbia astrachanica,* and *Heterocaryum echinophorum*).

**Figure 9:**
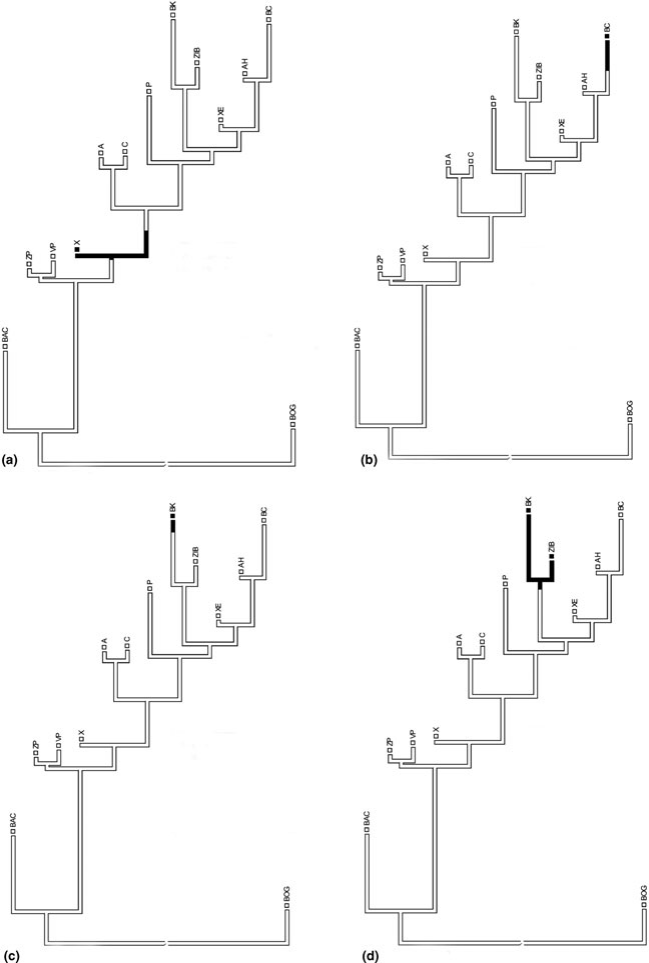
Species stochastic mapping reconstruction of selected species within the Lower Volga Valley: *Onosma setosa* (a), *Potamogeton skvortsovii* (b), *Puccinellia vitalii* (c), and *Melandrium astrachanicum* (d).

In some cases, dispersal and extinction events have not played a role in the current distribution of endemics (Fig. 8: *Astragalus baerii* (b), *Ceratophyllum kossinskyi* (c)*, Corispermum filifolium* (d)*;*Fig. 9: *Onosma setosa* (a)*, Potamogeton skvortsovii* (b)*, Puccinellia vitalii* (c)) or in a few others, dispersal has favored the expansion of species into adjacent areas with similar ecosystems, as in *Melandrium astrachanicum* (Fig. 9d).

Species stochastic reconstructions provide more in-depth scenarios that allow the generation of testable hypotheses for further investigation on species population structure and dynamics. For example, when we examine the history of *Crepis astrachanica* (Fig. 5b) we can see that extinction may have affected populations in the Blue-grey Hill (BK) area and extant populations may or may not have diverged from populations occurring in the neighboring Lakeland (ZIB). Similarly, new hypotheses on its current and past distribution along the river valley (areas XE, AH, and BC) can be generated and tested with population genetic studies.

Individual stochastic mapping reconstructions can be used to synthesize the formation of entire plant communities. For example, the *Suaedo salsae-Halocnemetum limonietosum suffruticosae* plant community was independently described by Golub and CČorbadze (1989) in Lakeland (ZIB) and Lysenko (unpubl. data) on Lake Baskunchak (BAC), and it includes *Halocnemum strobilaceum, Suaeda salsa, Climacoptera crassa, Petrosimonia brachiata, Limonium suffruticosum, Salicornia prostrata, Puccinellia fominii, Ceratocarpus arenarius, Chorispora tenella, Frankenia pulverulenta, Frankenia hirsuta,* and *Nitraria schoberi*, none endemic. Species stochastic reconstruction shows that dispersal and extinction have shaped the distribution of each of the above species in the LVV (e.g., Fig. 6b for *Suaeda salsa*). In time, these processes have led to the independent formation of the same plant community in two disjunct areas, clearly non-randomly.

## Conclusions

Given the complex geomorphological history of the LVV characterized by multiple tectonic events, including transgression and regression phases, the flora of the region has been shaped, often independently by its geomorphological history. Intuitively, we would have expected floristic elements to first occupy the northern areas that formed immediately after the last transgression phase. However, we found that geomorphologically older areas harbor floras that formed more recently. We recover a similar pattern by considering a hypothetical palaeoflora that may have been considerably smaller or larger than the extant flora. Our results show that the narrowly defined river valley and delta regions still harbor a more recent flora whilst adjacent drier habitats were the first to be colonized.

Overall, when examining stochastic mapping reconstructions, current species distributions have been independently shaped by dispersal, extinction, and in some cases vicariance events due to ecological or physical barriers (e.g., Baer knolls). Each species bears a unique history that may or not be under the direct influence of recent geological events, and may serve as the basis for further investigation on the evolution of local populations. Likewise, single species history can be used to synthesize the formation of whole plant communities and foster hypotheses on the ecological factors that provide stability and community structure.

Finally, our study shows that in the absence of taxon phylogenies and temporal information, species occurrence data may still be informative and analyzed using modern approaches. The results we generated represent testable hypotheses on the history of an area and its inhabiting flora.

## Supporting Information

**Appendix S1.** Nexus file with the list of 1018 species native to LVV and their occurrence coded on a standard matrix.
